# SynPoC: a high-quality generative diffusion model for transforming ultra-low-field point-of-care MRI using high-field MRI representations

**DOI:** 10.1038/s41598-025-33162-9

**Published:** 2026-01-24

**Authors:** Kh Tohidul Islam, Sanuwani Dayarathna, Shenjun Zhong, Parisa Zakavi, Helen Kavnoudias, Shawna Farquharson, Gail Durbridge, Hongfu Sun, Stephen Bacchi, Gary F. Egan, Markus Barth, Andrew Dwyer, Katie L. McMahon, Paul M. Parizel, Meng Law, Zhaolin Chen

**Affiliations:** 1 Monash Biomedical Imaging, Monash University, Blackburn Road, Clayton, VIC 3168 Australia; 2 Australian National Imaging Facility, Brisbane, QLD Australia; 3 Department of Neuroscience, Monash University, Clayton, VIC Australia; 4 Radiology, Alfred Hospital, Melbourne, VIC Australia; 5 Herston Imaging Research Facility, University of Queensland, Brisbane, QLD Australia; 6 School of Electrical Engineering and Computer Science, University of Queensland, Brisbane, QLD Australia; 7 South Australian Health and Medical Research Institute, Adelaide, SA Australia; 8 School of Clinical Science, Queensland University of Technology, Brisbane, QLD Australia; 9 David Hartley Chair of Radiology, Royal Perth Hospital, Perth, WA Australia; 10 Medical School, University of Western Australia, Perth, WA Australia; 11 Data Science and AI, Monash University, Exhibition Walk, Clayton, VIC 3800 Australia; 12 SA Medical Imaging, SA Health, Adelaide, SA Australia; 13 Surgery, Monash University, Clayton, VIC Australia; 14 Department of Neurology, Royal Adelaide Hospital, Adelaide, SA South Australia

**Keywords:** Brain imaging, Magnetic resonance imaging

## Abstract

Ultra-low-field (ULF) point-of-care (PoC) Magnetic Resonance Imaging (MRI) offers a promising pathway to improve accessibility in medical imaging due to its portability and lower cost. However, the diagnostic utility of ULF MRI is currently limited by lower image quality, particularly in signal-to-noise ratio, resolution, and contrast. To address this, we introduce SynPoC, a generative diffusion model designed to enhance ULF MRI by synthesizing high-field MRI-like images. SynPoC employs a conditional adversarial diffusion framework that leverages both noise and contrast-specific features to model inter-field representations. We evaluated SynPoC across a multi-site dataset of 180 participants, including both healthy individuals and patients with a variety of brain conditions. The enhanced images exhibited improved anatomical clarity and structural alignment with corresponding high-field MRI, as supported by quantitative and volumetric analyses. Our model demonstrates promise for image quality enhancement and research applications; however, as with other generative approaches, there is a non-zero risk of hallucinated or misleading features, particularly near low-SNR boundaries and fine structures. We therefore provide synchronized slice-by-slice comparison videos (3T, PoC, SynPoC) to aid reader inspection and emphasize that SynPoC is not intended for diagnostic decision-making without additional safeguards and validation. Further validation is warranted before diagnostic use.

## Introduction

In current clinical practice, Magnetic Resonance Imaging (MRI) relies on the use of superconducting high-field MRI technology, such as those operating at 1.5 Tesla (T) and 3.0T. However, high-field MRI systems are limited by their high installation, maintenance, and operational costs, which require specialized facilities and skilled workforce^1^. Consequently, access to these systems is predominantly restricted to well-resourced settings, creating a significant barrier to adoption in resource-limited environments and underserved regions^2–4^. This disparity in accessibility has become a growing concern, particularly as MRI’s role in clinical diagnostics continues to expand globally^5^.

Ultra-low-field (ULF) point of care (PoC) MRI presents a cost-effective alternative which can potentially increase accessibility in underserved regions^6–14^. These systems are more affordable, portable, and easier to maintain compared to their high-field counterparts, making them a promising solution for improving global healthcare equity^15,16^. To date, existing studies have successfully implemented key neuroimaging protocols for use with ULF scanners, demonstrating their clinical utility in detecting and diagnosing various brain lesions, including tumors and strokes^17–20^. Technological innovations, such as developing methods for actively detecting and removing external electromagnetic interference using analytical techniques and deep learning (DL) approaches, have also eliminated the requirement for radiofrequency shielded rooms^21,22^. These innovations have enabled the deployment of shielding-free ULF MRI scanners in PoC settings, such as intensive care units and emergency departments, expanding their use in diverse clinical environments^23^.

Despite their promise, PoC MRI technology faces fundamental challenges that impact their clinical utility^1,24,25^. A major limitation is the inherently lower signal-to-noise ratio (SNR) and image resolution compared to high-field MRI systems, which dramatically reduces the clinical value of these scanners and restricts their broader clinical adoption^26–28^. Additionally, while imaging contrast in ULF MRI depends heavily on the sequence type and acquisition parameters, the term ‘low contrast’ in this study specifically refers to the reduced ability of ULF MRI to distinguish subtle differences in tissue signal intensity^29^. This constraint arises from the fundamental physics of MRI, where SNR scales approximately with square of the field strength at higher field and the 7/4 power at low fields^30,31^. Consequently, the MR signal produced by ULF MRI is vastly weaker than that of high-field (3T) MRI^32,33^. Additionally, recent advanced image reconstruction methods are designed for high-field systems, which are not optimized for the lower signal environments of ULF MRI^31^. Although alternative computational techniques, have been explored (e.g. compressed sensing and MR fingerprinting) these methods have yet to fully resolve issues related to image quality and clinical applicability^34,35^.

Recent advances in DL provide opportunities to substantially improve image quality in medical imaging^36–38^. DL techniques, such as convolutional neural networks (CNNs) and generative adversarial networks (GANs), have been utilized to improve MRI image quality by reconstructing data from undersampled k-space or low-resolution images, effectively reducing noise and artifacts while enhancing resolution^39–45^. For instance, Lau et al.^42^ proposed a DL-based approach that synthesizes high-resolution images from low-field data by learning structural patterns from high-field datasets, while Lin et al.^43^ demonstrated a model that leverages multi-contrast images to enhance the quality of ULF MRI scans. Similarly, Man et al.^31^ reported the successful application of DL for artifact reduction and denoising in 3D brain MRI acquired at ultra-low-field strength (0.055 T), highlighting the effectiveness of combining advanced DL algorithms with innovative acquisition techniques to achieve fast, high-resolution imaging in environments with low SNR and prevalent artifacts. Iglesias et al.^46^ introduced SynthSR, a synthetic super-resolution (SR) method to convert low-resolution images into high-quality outputs suitable for clinical interpretation. A conditional GAN-based model was previously developed, significantly improving the image quality of ULF MRI through image-to-image translation using paired low- and high-field images^47^. Among the state-of-play generative artificial intelligence (GenAI) models, diffusion-based architectures have demonstrated unprecedented capability to model and synthesize medical imaging^38^. These models are highly capable of refining low-resolution medical images by modelling their fundamental data distributions^48–51^.

**Fig. 1:**
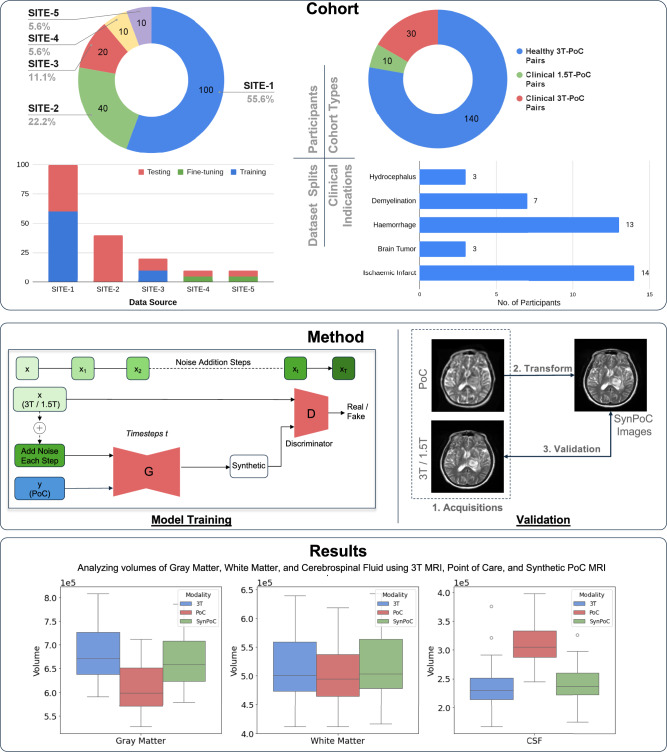
Demographic and clinical characteristics of participants. The study cohort included a total of 180 participants, with an age range from 18 to 81 years (mean age = 47.9 years, standard deviation = 17.9 years). The gender distribution comprised 86 males (47.8%) and 94 females (52.2%).

In this work, we introduce a novel adversarial diffusion-based generative model designed to transform PoC MRI images into high-field-like representations, called SynPoC (synthetic point of care) MRI. Inspired by the GAN architecture, SynPoC employs an adversarial diffusion setup to efficiently learn feature representations from both noise-degraded input and the corresponding conditioning MRI contrasts in a diffusion model. This design enables the extraction of contrast-specific information in the feature space, aiming to mitigate the contrast and SNR difference between MRI field strengths. Moreover, SynPoC is designed as a 2.5D model, leveraging adjacent 2D slices for efficient processing. While a fully 3D approach could potentially improve spatial consistency, it would require significantly higher computational resources, which may limit its practical application in ultra-low-field MRI datasets^52^. SynPoC is trained and validated using novel paired PoC and high-field MRI datasets, which include both healthy/normal individuals as well as patients representing a range of imaging scenarios, including pathological cases using multi-contrast MRI, including T1-weighted, T2-weighted, and Fluid-Attenuated Inversion Recovery (FLAIR). Our healthy datasets are from Monash Biomedical Imaging (SITE-1) and Herston Imaging Research Facility (SITE-2), whereas the clinical datasets are from the Alfred Hospital (SITE-3), the Royal Perth Hospital (SITE-4), and the South Australian Health and Medical Research Institute at the Royal Adelaide Hospital (SITE-5). Figure 1 provides an overview of the study, including demographic and clinical information, model training and validation strategy. Human research ethics approval was obtained and informed consent was acquired in writing from all participants or their representatives prior to inclusion in the study. Our data acquisition spans from October 2022 to April 2024, resulting in a cohort of 180 paired high-field and PoC datasets, with participants scanned on both systems at the same time point. While diffusion models can substantially improve perceived image quality, they may also introduce artifacts or hallucinated structures when inputs are noisy, undersampled, or out-of-distribution. In the context of ULF MRI, this risk is most pertinent along tissue interfaces (e.g., CSF–parenchyma), small nuclei, and lesion borders. To make these limitations transparent and to support critical assessment by readers, we include synchronized slice-by-slice comparison videos (3T reference, native PoC, and SynPoC) for all critical examples, and we frame hallucination mitigation and uncertainty communication as key priorities for future work.

## Results

Qualitative and quantitative results of the SynPoC model for improving PoC MRI images are shown.

### Model output images

**Fig. 2:**
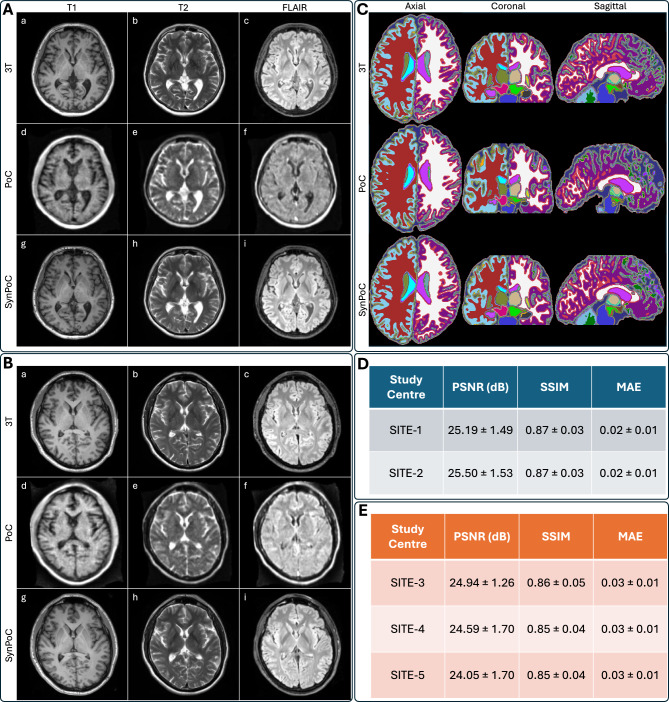
Qualitative and quantitative results from different MRI modalities across imaging sites and healthy and clinical participants. (**A**) Results for a healthy male participant in his early 60s from imaging site SITE-1. (a)–(c) represent 3T T1, T2, and FLAIR sequences, (d)–(f) show corresponding PoC sequences, and (g)–(i) illustrate SynPoC images. (**B**) Results for a healthy female participant in her late 40s from imaging site SITE-2 (Herston Imaging Research Facility). (a)–(c) represent 3T T1, T2, and FLAIR sequences, (d)–(f) show corresponding PoC sequences, and (g)–(i) illustrate SynPoC images. (**C**) Brain parcellation volumes across different MRI modalities from imaging site SITE-1 (male, late 30s). Each column represents a different view in axial, coronal, and sagittal orientations, with rows corresponding to the respective MRI modalities. (**D**) Quantitative performance metrics (PSNR, SSIM, MAE) for healthy data from SITE-1 and SITE-2. (**E**) Quantitative performance metrics for clinical data from SITE-3, SITE-4, and SITE-5.

Figure 2 presents qualitative and quantitative comparisons of MRI modalities across different sites, including brain parcellation and performance metrics for both healthy and clinical participants. SynPoC demonstrates notable improvements in visual clarity, anatomical detail, and resemblance to high-field MRI images. Figure 2A illustrates the image output of the SynPoC model applied to ULF MRI data from a healthy participant at SITE-1. Whereas the ULF T1-weighted MRI images exhibit limited anatomy in the cortical gyration and deep gray nuclei, these features appear markedly improved in the synthetic SynPoC images, though some fine structures may still differ from true high-field anatomy. Similarly, ULF FLAIR images show significantly lower contrast between gray and white matter, making it difficult to distinguish these regions. SynPoC enhances the contrast, yielding FLAIR images with improved differentiation between gray and white matter that more closely resembles high-field MRI, albeit with subtle alterations in contrast representation. Across all sequences, the ventricular Cerebrospinal fluid (CSF) spaces appear larger at ULF than the reference 3T, which is visually adjusted by SynPoC toward 3T-like appearance. Visual resemblance between SynPoC and 3T is also observed in extracranial structures such as the skull and scalp. Similar results are achieved from SITE-2, Figure 2B, which is noteworthy as this center did not contribute data to the training model. Brain parcellations using the SynthSeg${^+}$^53^ and the MGH-subcortical atlas, as shown in Fig. 2C, indicate that SynPoC-generated images support improved structural delineation of subcortical anatomy, showing high similarity to 3T images in appearance.

### Quantitative image metrics and inter-site variability

Commonly used image quality metrics, including Peak Signal-to-Noise Ratio (PSNR), Structural Similarity Index Measurement (SSIM), and Mean Absolute Error (MAE) for SynPoC images versus the high-field reference for the healthy group datasets, highlighting how the model adjusts ULF image characteristics to better resemble high-field representations, are shown in Fig. 2D^54^. While these metrics offer a quantitative perspective on image similarity, they are inherently limited in capturing clinical relevance or perceptual quality, as highlighted in previous studies^55^. To address this, we additionally assessed volumetric consistency in brain regions commonly examined in neuroimaging studies. Data from SITE-1, used for primary model development, and SITE-2 showed no statistically significant between-site difference in any of PSNR (t = −0.80, *p* = 0.430), SSIM (t = 0.00, *p* = 1.000), or MAE (t = 0.00, *p* = 1.000), with no statistically significant differences in structural similarity metrics. These quality metrics also remained consistent across clinical sites, SITE-3 to SITE-5, in patients with various clinical imaging scenarios (Fig. 2E). Data from SITE-3, SITE-4, and SITE-5 showed no statistically significant differences in PSNR or SSIM between sites. The marginal differences in the quantitative metrics between healthy and clinical sites are likely attributed to the domain shifts due to clinical image features.

**Fig. 3:**
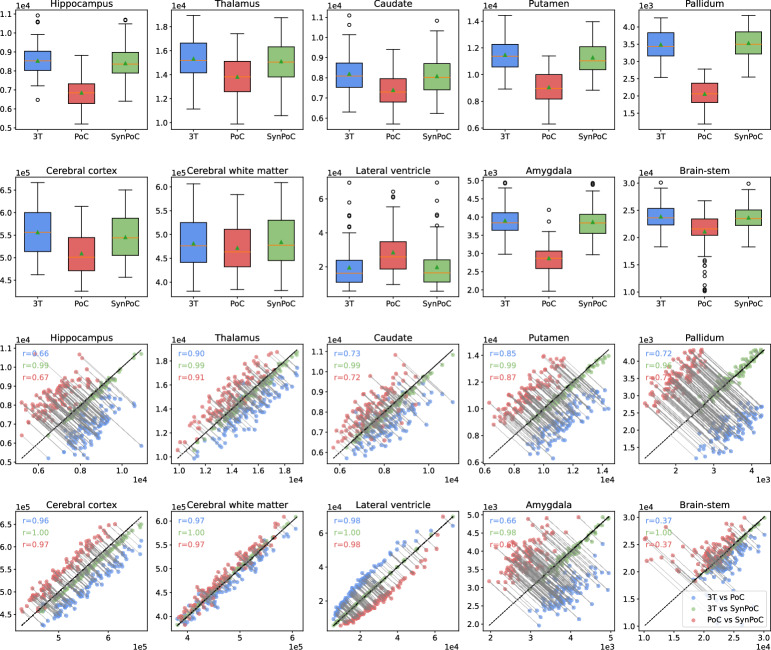
Volumetric measurements (cubic mm) of key brain regions across different MRI modalities for the healthy dataset, with boxplots showing the distribution of measurements and scatter plots illustrating the correlation between modalities.

Ten brain regions analyzed in the healthy cohort are shown in Fig. 3, selected due to their frequent evaluation in neuroimaging studies of neurological disorders. Volumetric changes in these regions could indicate disease progression or severity. The median and interquartile range show SynPoC shows closer volumetric agreement with 3T measurements, particularly in the hippocampus, thalamus, and caudate, with a high level of agreement with 3T data. The scatter plots show strong correlation between measurements from SynPoC and 3T compared to only moderate between 3T and PoC in hippocampus (3T vs. SynPoC: r = 0.99 vs. 3T vs. PoC: r = 0.66) and caudate (3T vs. SynPoC: r = 0.99 vs. 3T vs. PoC: r = 0.73). Improvements were also seen in the thalamus (3T vs. SynPoC: r = 0.99 vs. 3T vs. PoC: r = 090) and cerebral cortex (3T vs. SynPoC: r = 1.00 vs. 3T vs. PoC: r = 0.96). Where the correlation between 3T and PoC is weak such as the brainstem (r = 0.37), the SynPoC markedly improves volumetric correlation with the 3T data (3T vs. SynPoC: r = 1.00). Volumetric measurements of total gray matter (GM) and white matter (WM) showed no significant between-site difference comparing SITE-1 with SITE-2 across any data series. A significant difference in total CSF volume was observed between SITE-1 and SITE-2 in both 3T (p = 0.034) and SynPoC (p = 0.038) datasets. However, this difference was not detected in native PoC images (*p* = 0.138), suggesting that SynPoC preserves inter-site variability patterns more similarly to 3T than native PoC images.

### Model application to disease

We further explored the application of SynPoC on clinical imaging datasets to assess image enhancement in pathological cases. The image results were reviewed by three board-certified radiologists (A.D., P.M.P., M.L.), each with more than 20 years’ experience in neuroimaging. Quantitative analysis was subsequently performed.

#### Hydrocephalus

**Fig. 4:**
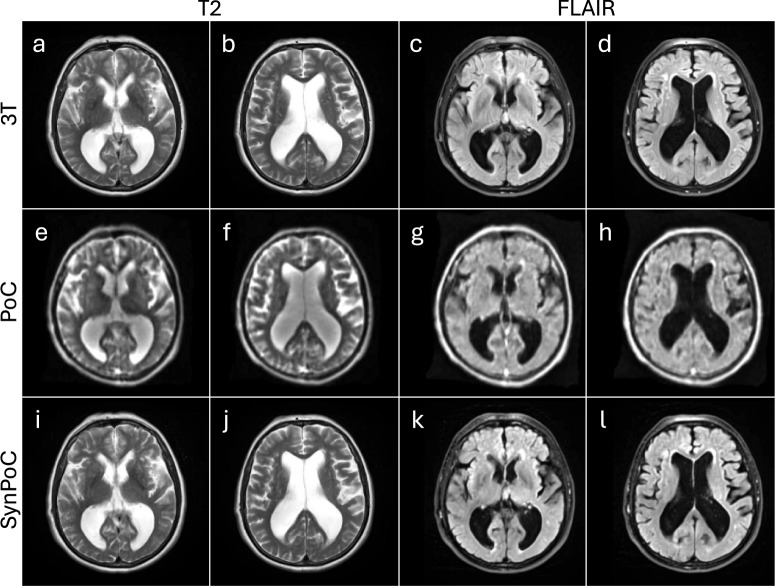
Imaging results for a male patient in his 70s diagnosed with Normal Pressure Hydrocephalus (NPH) from site SITE-3 (the Alfred Hospital). (**a**) and (**b**) 3T T2 images, (**c**) and (**d**) 3T FLAIR images, (**e**) and (**f**) PoC T2 images, (**g**) and (**h**) PoC FLAIR images, (**i**) and (**j**) SynPoC T2 images, and (**k**) and (**l**) SynPoC FLAIR images.

Figure 4 shows an MRI study of a male patient in his 70s diagnosed with normal pressure hydrocephalus (NPH). 3T images show ventriculomegaly and periventricular T2 hyperintensity indicating transependymal edema. ULF T2-weighted images also depict ventricular enlargement, but periventricular hyperintensity is less clearly depicted. SynPoC images show refinements in image quality, with enhanced depiction of periventricular hyperintensity relative to native PoC images. The SynPoC FLAIR images similarly exhibit enhanced detail, visually approaching that of the 3T FLAIR, though subtle differences remain. Lateral ventricular volume (from T2) was 219682 mm$\vphantom{0}^3$ (219.7 mL) at 3T, 182002 mm$\vphantom{0}^3$ (182.0 mL) at PoC and 222268 mm$\vphantom{0}^3$ (222.3 mL) at SynPoC indicating SynPoC volumetric estimates were numerically closer to those from 3T, though these results should be interpreted with caution as the synthetic process may introduce structural biases not present in native images.

#### Demyelination

**Fig. 5:**
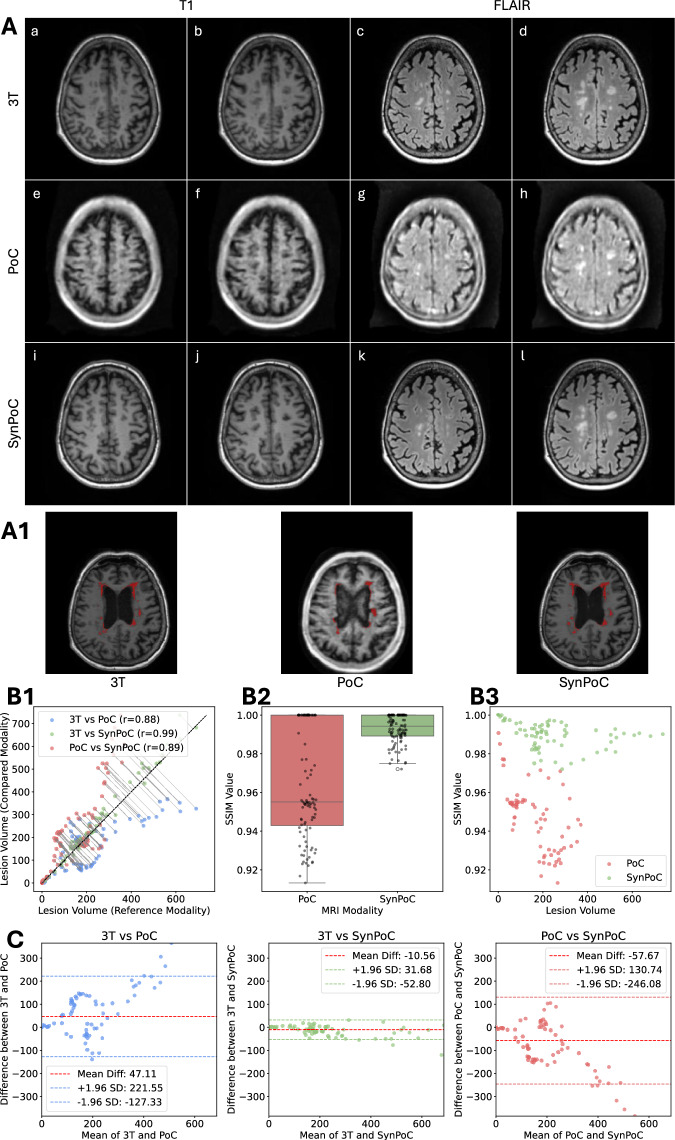
Imaging and lesion analysis for a female patient in her 70s diagnosed with Multiple Sclerosis (MS) from site SITE-3. (**A**) Imaging results comparing 3T T1 and FLAIR sequences with PoC and SynPoC images. (a) and (b) are 3T T1 images, (c) and (d) are 3T FLAIR images, (e)–(h) are corresponding PoC images, and (i)–(l) are SynPoC images. (**A1**, **B1**, **B2**, **B3**, and **C**) Analysis of MS lesion characteristics across different MRI modalities (3T, PoC, and SynPoC), demonstrating variations in lesion presentation and comparison of imaging results.

Figure 5 shows imaging results for Multiple Sclerosis (MS) in SITE-3, comparing 3T, PoC, and SynPoC modalities, along with an analysis of MS lesions across different clinical sites. Figure 5A presents a female patient in her 70s with known MS. T1-weighted images at 3T, Figure 5A(a) and (b), demonstrate multiple hypointense periventricular white matter lesions characteristic of MS with high clarity. ULF images, Figure 5A(e) and (f), also reveal the presence of periventricular lesions but appear larger and less defined, likely due to partial volume effects from reduced resolution and contrast. The SynPoC images in Fig. 5A(i) and (j) show improvements in lesion visibility and anatomical detail, visually approximating features seen in high-field images. Figure 5A(c) and (d), and (g) and (h), show FLAIR images at 3T and ULF, respectively. In high-field FLAIR images, the clinical standard-of-care for assessing lesion burden, the demyelinating plaques are more conspicuous than those at ULF, with clearer demarcation from surrounding white matter.

SynPoC FLAIR images (Fig. 5A(k) and (l)) also show improved clarity and lesion definition, suggesting potential for supporting multi-sequence visual analysis, though their use in clinical decision-making requires further investigation. We further conducted an analysis of MS lesion volume across patients from SITE-3 using contrast-adaptive multimodal segmentation tools^56^, as shown in Fig. 5A1. Figure 5B1 shows a high correlation (r = 0.99) between SynPoC and 3T images, along with a clear improvement in lesion volume estimates over the native ULF images, though some synthetic bias may still exist.

SynPoC scores higher SSIM values than ULF, reflecting improved structural similarity to 3T images in lesion regions; however, SSIM does not fully capture clinical accuracy or diagnostic relevance. This performance is maintained across a range of lesion sizes and total lesion burden (Fig. 5B3). Bland-Altman analysis (Fig. 5C) evaluates the agreement between lesion volume measurements across different modalities: 3T vs. PoC, 3T vs. SynPoC, and PoC vs. SynPoC. SynPoC shows narrower limits of agreement and smaller mean differences compared to PoC, indicating better volumetric alignment with 3T; however, care is needed when interpreting synthetic lesion metrics. These results suggest SynPoC-enhanced images may support longitudinal visual comparison with 3T, though prospective validation and caution are required.

#### Ischaemic infarct

**Fig. 6:**
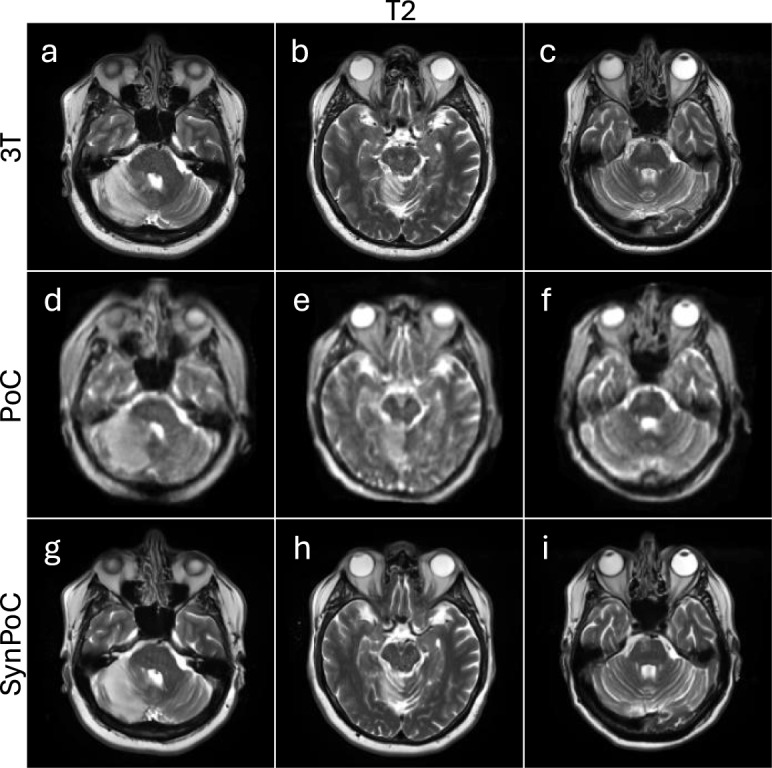
Imaging results for three male patients in their early 70s and late 60s from site SITE-5 with acute ischaemic infarcts in different brain regions. (**a**)–(**c**) 3T, (**d**)–(**f**) PoC, and (**g**)–(**i**) SynPoC. No data from SITE-5 were used to train the model, but fine-tuning was done.

Figure 6 shows three patients with acute ischaemic infarcts in different brain regions, a male in his early 70s diagnosed with a large middle cerebral artery cerebral infarct, a male patient in his late 60s diagnosed with an acute right superior cerebellar artery infarct and a male patient in his late 60s diagnosed with a right pontine brainstem infarct (Fig.  6). Figure 6a–c display 3T T2-weighted images providing high-resolution visualization of the infarcted area. These images capture detailed morphological features, including the extent of the infarct and associated edema, which are crucial for accurate diagnosis and management of ischemic stroke.

The ULF MRI images (Fig. 6d–f) identify the presence of the infarct but with diminished signal, resolution and contrast. The boundaries of the infarct are less defined and in smaller infarcts, the lower SNR may contribute to diagnostic error. The SynPoC images (Fig. 6g–i) exhibit improvements in visual quality, enhancing infarct visibility and more closely approximating the clarity and structure seen in 3T MRI images. These visual enhancements suggest that SynPoC may recover structural detail less apparent in native PoC images; however, caution is required when interpreting synthetic images in a diagnostic context. This visual trend was observed across infarcts of varying sizes, but further validation is needed to confirm generalizability.

#### Haemorrhage

**Fig. 7:**
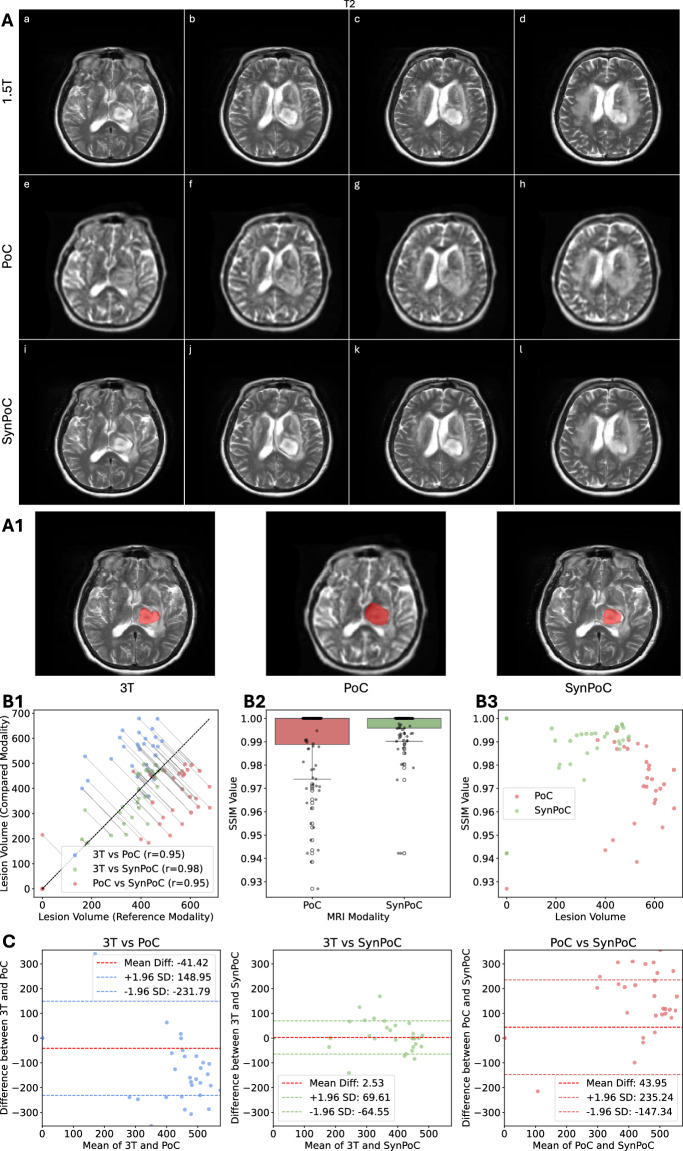
Imaging results for a female patient in her late 70s diagnosed with Left Thalamic Hemorrhage with Intraventricular Extension from SITE-4 (The Royal Perth Hospital). (**A**) Imaging results comparing 1.5T T1 and T2 sequences with PoC and SynPoC images. (a)–(b) 1.5T T2 images, (c)–(d) 1.5T T1 images, (e)–(f) PoC T2 images, (g)–(h) PoC T1 images, and (i)–(j) SynPoC T2 images, (k)–(l) SynPoC T1 images. Initially, no data from SITE-4 were used to train the model, and 3T data were not part of their clinical workflow. The model was subsequently fine-tuned using data from SITE-4. (**A1**, **B1**, **B2**, **B3**, and **C**) Analysis of hemorrhage lesions across 3T, PoC, and SynPoC MRI modalities.

Figure 7 presents imaging results for a hemorrhage case from SITE-4, comparing 1.5T, PoC, and SynPoC modalities, along with an analysis of hemorrhage lesions across different MRI sequences. Figure 7A provides a comparative analysis of MRI scans for a female patient in her late 70s diagnosed with left thalamic hemorrhage and intraventricular extension from study SITE-4. T2-weighted images from a 1.5T MRI system demonstrate T2 hyperinense haemorrhage, likely late subacute methaemoglobin phase, a thin rim of lower T2 signal, adjacent oedema and mass effect.

ULF MRI images are able to identify the main features but return different internal signal and do not resolve the more detailed rim features or intraventicular extension which could affect patient management. The SynPoC images exhibit enhanced visualization of the haemorrhagic lesion, with improved delineation of margins and surrounding structures. These improvements suggest that SynPoC may enhance the visibility of features less apparent in PoC images, though care is warranted in interpreting synthetic detail as diagnostic truth. Lesion quantification, Figure 7A1, B1, B2, B3, C, shows high volumetric correlation between SynPoC and 3T, whereas PoC images tend to underestimate volume. However, the possibility of synthetic bias or hallucination should not be overlooked.

#### Clinical brain morphometry

**Fig. 8:**
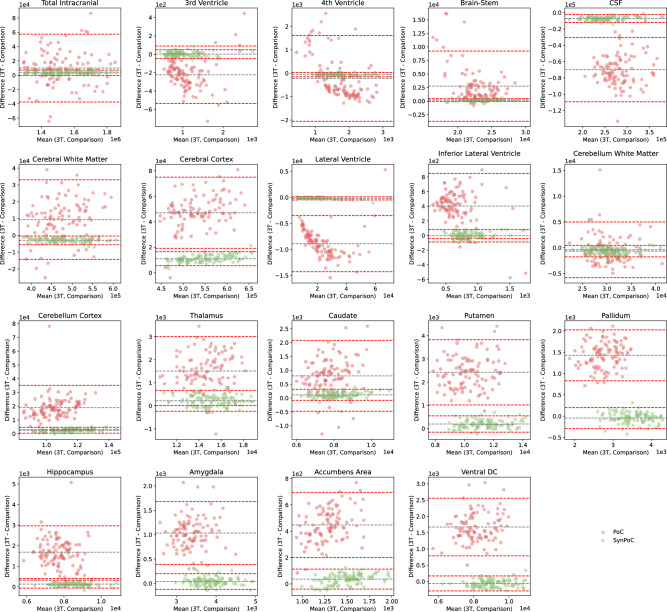
Bland-Altman plots demonstrating the agreement between volumetric measurements from high-field MRI and those derived from PoC and SynPoC MRI images. Each subplot corresponds to a different brain region, illustrating the mean differences and limits of agreement.

Agreement and bias of regional brain volumes in key brain regions, segmented using the SynthSeg${^+}$^53^ model, are shown in Bland-Altman plots for PoC and SynPoC images compared with high-field MRI (Fig. 8). Measurement of regional brain structure plays a critical role in research and clinical assessment of neurodegenerative processes. However, inaccuracies in volume estimates can influence interpretation in clinical and research contexts. Total intracranial volume showed a minimal mean difference between SynPoC and high-field images, suggesting improved alignment in some global anatomical measurements; however, these outputs remain synthetic and should be interpreted with caution. However, wider limits of agreement in regions such as the Amygdala and Hippocampus highlight challenges in achieving consistent enhancement in anatomically variable or clinically sensitive regions.

## Discussion

This study presents SynPoC, a diffusion-based generative AI (GenAI) model developed to generate ultra-low-field (ULF) point-of-care (PoC) MRI images by synthesizing high-field-like representations. The model demonstrates consistent improvements in visual clarity, anatomical and pathological feature depiction, and structural alignment across multiple imaging sites and diverse participant cohorts. The SynPoC model targets key limitations of ULF MRI systems, including low SNR, limited contrast, and reduced spatial resolution. By synthesizing high-field-like representations, the model improves alignment with standard MRI features, offering a potential visual enhancement over native ULF images. These enhancements were observed across a range of imaging scenarios, including healthy participants and individuals with diverse brain pathologies. The model also demonstrated visual consistency across datasets from multiple imaging centers, suggesting a degree of generalizability. These promising results warrant further validation in clinical settings.

Many methods have been developed to improve different aspects of the ULF image quality, including improving image resolution^42^, enhancing image contrasts^46^, and reducing image artifacts^31^. Compared with the existing methods, SynPoC introduces a diffusion-based framework that enables progressive refinement of the ULF MRI images while preserving anatomical consistency with reference to high-field MRI. Notably, SynPoC is trained using real paired ULF and high-field datasets, rather than relying solely on simulated inputs as in prior studies^31,42,46^, which may enhance realism and generalizability in specific domains. Nonetheless, we acknowledge that image-to-image translation approaches may alter the native signal content of MRI scans. Therefore, careful validation, including radiological review and uncertainty quantification, is necessary before considering any diagnostic or longitudinal use.

The pathological datasets used in this study have explored the visual representation of lesions related to acute haemorrhage, ischaemic stroke, demyelination, and hydrocephalus using SynPoC-enhanced images. While the synthetic images provided enhanced lesion visibility in certain cases, caution is warranted as these improvements do not necessarily translate to diagnostic accuracy. The ability to generate visually enhanced images from portable, low-cost MRI systems may support image interpretation in environments where access to high-field MRI is limited, such as acute care triage and remote settings. Quantitative comparisons with 3T imaging across cortical, subcortical, and lesion regions suggest that SynPoC may support certain research tasks such as volumetric estimation and morphometric analyses. However, it is important to recognize that metrics such as SSIM and PSNR primarily assess pixel-level similarity and do not directly correspond to diagnostic performance or clinical reliability. In this study, we supplemented standard metrics with volumetric consistency checks to provide a more robust and clinically contextual evaluation of the model’s performance. These supplementary analyses provide a broader perspective on the model’s behavior, though additional validation is needed to assess alignment with clinical decision-making standards.

The prospect of leveraging data-enhanced ULF MRI to support neuroimaging in resource-limited environments is particularly compelling given the logistical and financial challenges associated with high-field systems, and the need for bedside imaging in high-acuity hospital settings. Additionally, this study highlights the potential of diffusion-based models to enhance image quality in low-field settings, contributing to the broader body of research on deep learning in medical imaging. The integration of generative AI with medical imaging technologies may offer new opportunities to improve image interpretability and accessibility, though rigorous validation and careful clinical consideration remain essential.

However, several limitations must be acknowledged when considering the potential clinical relevance of the SynPoC model. First, the model’s generalizability may be constrained by limited diversity in the training data. While SynPoC produced promising results across the datasets used in this study, its efficacy on images acquired with different protocols, scanners, or from populations with varying characteristics remains uncertain. The model’s ability to generalize to out-of-distribution applications, such as detecting abnormalities not included in the training set, is a critical concern. This limitation was highlighted in this study by the mixed performance observed when evaluating a single brain tumor, which was present only in the validation cohort. When presented with unfamiliar pathologies or anatomical variations, the model may produce degraded or misleading outputs, raising concerns about potential diagnostic inaccuracies or hallucinated features.

Second, a key concern with generative models like SynPoC is model uncertainty and the risk of hallucinated or misleading outputs. Diffusion-based GenAI models may introduce artifacts, distort structural features, or insert anatomically implausible details into synthesized images, particularly when processing input data with low SNR or noise-related degradation. This presents a significant risk in clinical contexts, where artificial features may be misinterpreted as true pathology or obscure relevant findings. For example, SynPoC may consistently generate features such as vascular flow voids based on patterns learned during training, even in cases where such features are absent due to pathology. In such scenarios, such as vascular occlusion or other pathology-driven changes, the model may erroneously recreate expected features, potentially resulting in false negatives or misdiagnoses. Moreover, anatomical regions with high inter-individual variability, such as cortical folds or small subcortical nuclei, may be particularly susceptible to inconsistent or inaccurate reconstruction.

The performance of SynPoC is inherently dependent on the diversity and representativeness of its training data. Although the generated outputs show improved alignment with high-field features, the generation process may inadvertently alter tissue contrast and structural integrity. This raises important concerns about the interpretability of SynPoC-enhanced images, particularly in clinical scenarios that require accurate tissue characterization for differential diagnosis. While SynPoC appears to capture individual variability to some extent, as observed in consistent outputs among healthy participants across sites, it may perform more reliably on anatomical structures that exhibit low inter-subject variability. Conversely, regions with high anatomical variability or rare pathologies may require substantially more diverse training data to achieve comparable fidelity and reliability. This limitation could compromise the model’s ability to detect or accurately characterize abnormalities that were underrepresented or entirely absent during training. Although the model demonstrates promising visual and quantitative performance on the studied dataset, the possibility of hallucinations or misleading outputs cannot be ruled out. This underscores the need for cautious interpretation of AI-enhanced images and highlights the importance of incorporating uncertainty quantification to better inform clinical trust and decision-making.

Third, SynPoC’s reliance on site-specific fine-tuning to accommodate domain shifts, such as variations in imaging protocols or the presence of uncommon pathologies, may limit its scalability and generalizability across diverse clinical environments. Additionally, operational constraints, including the high computational demands for training and inference, and the requirement for standardized imaging protocols, pose practical challenges for implementing SynPoC in real-world workflows. In particular, the high computational load of AI may restrict the feasibility of near real-time enhancement, especially in low-resource or remote settings where PoC MRI is most needed.

Future work should focus on expanding the training dataset to include a broader spectrum of pathologies, anatomical variability, and acquisition protocols to improve the model’s generalizability and robustness. Incorporating mechanisms to quantify and communicate model uncertainty will be critical for mitigating the risks of hallucinated or misleading features in synthesized images. While the current 2.5D implementation provides a practical trade-off between performance and computational efficiency, investigating a fully 3D architecture could further improve volumetric consistency and spatial fidelity. Finally, rigorous external validation across diverse populations and imaging settings will be essential to assess the safety, reliability, and clinical suitability of AI-enhanced imaging techniques such as SynPoC.

Generative enhancement carries an intrinsic risk of hallucinated or misleading features. In ULF settings, this risk is amplified by low SNR and domain shifts. During qualitative review, we noted three recurring patterns that warrant caution: (i) boundary regularization at CSF–parenchyma interfaces; (ii) over-sharpening of fine textures (e.g., cortical micro-patterns) that may not reflect native signal; and (iii) lesion-edge smoothing that could obscure subtle rims or satellite foci. Integrating GenAI-generated medical images into clinical practice remains a significant challenge. Radiologists are likely to remain cautious when interpreting AI-enhanced images, particularly due to concerns about the authenticity, traceability, and reliability of reconstructed features. The introduction of AI-generated content in diagnostic workflows necessitates transparency in both model operations and rigorous validation of outputs. Enhancing model explainability, clearly communicating the purpose and limitations of image refinement, and incorporating uncertainty estimates could foster greater trust among clinicians by supporting informed interpretation of AI-generated images. Engagement with the radiological community will be essential to address concerns about clinical interpretation, image authenticity, and accountability. Collaborative efforts to develop consensus guidelines, best practices, and regulatory frameworks will be critical to ensuring the safe and appropriate use of generative AI models in clinical imaging. These observations, together with the quantitative improvements, underscore the need to treat SynPoC as an investigational research tool rather than a diagnostic substitute.

In conclusion, the SynPoC model demonstrates potential for refining ULF MRI images into representations that resemble high-field acquisitions, addressing some technical barriers to improved image quality in PoC imaging. However, careful consideration of its limitations, and those of generative AI models more broadly, is essential. Addressing these challenges through continued research, validation, and multidisciplinary collaboration will be key to responsibly advancing this technology toward future clinical applicability and broader accessibility in healthcare imaging.

## Materials and methods

All experiments were performed in accordance with relevant guidelines and regulations, and this study was approved by the Monash University Human Research Ethics Committee (MUHREC) (approval no. 32631).

### Sequence protocol

Table 1 summarizes the detailed imaging parameters for the MRI systems and sequences used in this study across multiple sites. The study utilized the Siemens Skyra 3T system (SITE-1, SITE-2, SITE-3, and SITE-5), Siemens MAGNETOM Sola Fit 1.5T system (SITE-4), and the ULF Hyperfine Swoop (64mT PoC). The parameters listed include voxel resolution, matrix size, repetition time (TR), echo time (TE), inversion time (TI), and acquisition duration for T1-weighted, T2-weighted, and FLAIR sequences.

**Table 1:** Detailed imaging parameters of MRI systems and sequences used in the study.

System	Sequence	FoV (mm)	Acquisition matrix	TR (ms)	TE (ms)	TI (ms)	Duration (min)
3T	T1	256 $\times$ 256 $\times$ 176	256 $\times$ 256 $\times$ 176	2100	2.21	1100	5.30
	T2	220 $\times$ 220 $\times$ 116	512 $\times$ 461 $\times$ 29	8000	102	2370	1.50
	FLAIR	232 $\times$ 256 $\times$ 176	232 $\times$ 256 $\times$ 176	5000	388	1600	5.40
1.5T	T1	208 $\times$ 256 $\times$ 176	256 $\times$ 208 $\times$ 176	2200	3.03	900	2.60
	T2	201 $\times$ 229 $\times$ 120	415 $\times$ 291 $\times$ 30	6000	89	–	2.60
PoC	T1	220 $\times$ 180 $\times$ 180	112 $\times$ 112 $\times$ 36	1500	6.62	300	6.17
	T2	220 $\times$ 180 $\times$ 180	146 $\times$ 146 $\times$ 36	2000	208.4	–	6.30
	FLAIR	220 $\times$ 180 $\times$ 180	138 $\times$ 138 $\times$ 36	4000	199.6	1402	9.30

### Multi-site fine-tune and validation datasets

To comprehensively evaluate the model’s generalizability and performance, multi-site validation was conducted using datasets from SITE-1 to SITE-5, including both healthy and clinical cohorts. For the healthy datasets, SITE-1 contributed 60 cases for training and 40 cases for testing, while SITE-2 provided 40 cases for testing. In the clinical cohorts, SITE-3 supplied 10 cases for training and 10 cases for testing, all involving patients with conditions such as MS and hemorrhage. Most datasets from these sites were acquired using 3T and PoC MRI systems, except for SITE-4, which used both 1.5T and PoC MRI for fine-tuning with 5 cases. Additionally, SITE-5 contributed 5 cases for fine-tuning, also using 3T and PoC MRI systems. This multi-site protocol ensured a robust assessment of the model’s adaptability and performance across varied technological settings and clinical environments. All experiments were performed in accordance with relevant guidelines and regulations. This study was approved by the Monash University Human Research Ethics Committee (approval no. 32631).

### Data quality control and pre-processing

Several key operational procedures were implemented to ensure the reliability and consistency of MRI data across multiple sites. Phantom calibration was performed regularly at each site to maintain imaging accuracy and consistency across different MRI systems. Efforts were made to minimize electromagnetic interference (EMI), particularly when using the Hyperfine Swoop in unshielded environments. In instances where EMI was detected, prompt action was taken to reposition the scanner to mitigate these concerns, ensuring uninterrupted data collection. Standardized patient positioning protocols were established to maintain consistency in image acquisition, reducing variability across different sites. Additionally, all datasets underwent rigorous preprocessing steps, including bias field correction (FSL-FAST) and spatial normalization (1 cubic millimeter isotropic resolution), and co-registration of the ULF and high-field scans via FMRIB’s Linear Image Registration Tool (FLIRT) to ensure uniform data quality and structure^57^. These measures were critical in maintaining the integrity of the data collected from diverse clinical environments. By adhering to these multisite operational procedures, the study ensured that the data used for training and testing the developed models was of high quality and consistency. This approach provided critical insights into the model’s effectiveness in real-world clinical applications, highlighting its potential utility and limitations.

To facilitate careful evaluation of potential synthetic bias or hallucinated features, we provide synchronized axial videos for each critical example, displaying 3T, PoC, and SynPoC volumes side-by-side. Readers are encouraged to verify: (i) spatial alignment of sulcal/gyral patterns and ventricular contours; (ii) lesion borders and perilesional signal; and (iii) small, high-contrast structures (e.g., deep gray nuclei) for over-sharpening or over-regularization. We explicitly caution that SynPoC outputs are investigational research images and should not be used for clinical decision-making without independent validation.

### The SynPoC model

The SynPoC model leverages an adversarial diffusion-based approach for synthesizing high-field MRI from ultra-low-field MRI contrast. We use a novel feature-based approach to adversarially train the denoising process on feature representations from both ultra-low and high-field MRI contrasts. This enables the identification of contrast-specific features, allowing the denoising process to map the differences between field strengths more accurately. The diffusion model consists of two main processes: the forward diffusion process and the reverse diffusion process^58^.

#### Forward diffusion process

The forward process involves gradually adding Gaussian noise at different time steps $t$ to the original high-field imaging until it becomes complete Gaussian noise, as described in Eq. (1):

1 $$\begin{aligned} \begin{aligned} q(x_t | x_{t-1}) = \mathcal {N} (x_t;\sqrt{1-\beta _t}x_{t-1}, \beta _t I) \end{aligned} \end{aligned}$$

where $\beta _t$ is the noise variance schedule, $\mathcal {N}$ denotes the Gaussian distribution, and $I$ is the identity covariance matrix.

#### Reverse diffusion process

The true denoising distribution guided by ULF MRI contrast $y$, $q(x_{t-1}|x_t,y)$, can be derived from the forward process, as in (Eq. 2):

2 $$\begin{aligned} q(x_{t-1}|x_t,y) = \mathcal {N}(x_{t-1}; \tilde{\mu }(x_t, t), \tilde{\beta }_t I), \end{aligned}$$

where:

$$\begin{aligned} \tilde{\mu }(x_t, t) = \frac{\sqrt{\alpha _{t-1}}\beta _t}{1-\alpha _t} x_0 + \frac{\sqrt{\alpha _t}(1-\alpha _{t-1})}{1-\alpha _t} x_t, \end{aligned}$$

$$\begin{aligned} \tilde{\beta }_t = \frac{1-\alpha _{t-1}}{1-\alpha _t} \beta _t. \end{aligned}$$

Here, $\alpha _t = \prod _{i=1}^t (1 - \beta _i)$ represents the cumulative noise scaling factor. This posterior represents the true distribution at each timestep $t-1$, conditioned on the noisy image $x_t$.

The reverse denoising process $p$ can be parameterized to recover the actual high-field image from the noise by estimating the true denoising distribution, guided by the conditional ULF MRI contrast $y$, as described in Eqs. (3) and (4):

3 $$\begin{aligned} \begin{aligned} p_{\theta }(x_{0:T}|y) = p(x_T)\prod _{t\geqslant 1}^{} p_\theta (x_{t-1} | x_t, y) \end{aligned} \end{aligned}$$

4 $$\begin{aligned} \begin{aligned} p_\theta (x_{t-1}|x_t, y) = \mathcal {N} (x_{t-1}; \mu _\theta (x_t, y, t), \sigma ^2_\theta (x_t, y, t)), \end{aligned} \end{aligned}$$

where $\mu _\theta (x_t, y, t)$ and $\sigma ^2_\theta (x_t, y, t)$ are the predicted mean and variance of the distribution, respectively. These are modeled by a neural network $\theta$ trained to maximize the evidence lower bound (ELBO) by matching the actual denoising distribution $q(x_{t-1}|x_t, y)$ with the parameterized model $p_\theta (x_{t-1}|x_t, y)$.

Instead of making a direct prediction on $x_{t-1}$, the denoising models can be interpreted as parameterized models as follows in Eq. (5).

5 $$\begin{aligned} \begin{aligned} p_\theta (x_{t-1}|x_t, y)\sim q(x_{t-1}|x_t, x_0 = G_\theta (x_t, y, t)) \end{aligned} \end{aligned}$$

where $x_0$ is predicted using the denoising model $G_\theta (x_t, y, t)$, and $x_{t-1}$ is predicted using the posterior distribution $q(x_{t-1}|x_t, x_0)$^58^.

#### Adversarial diffusion model approach

**Fig. 9:**
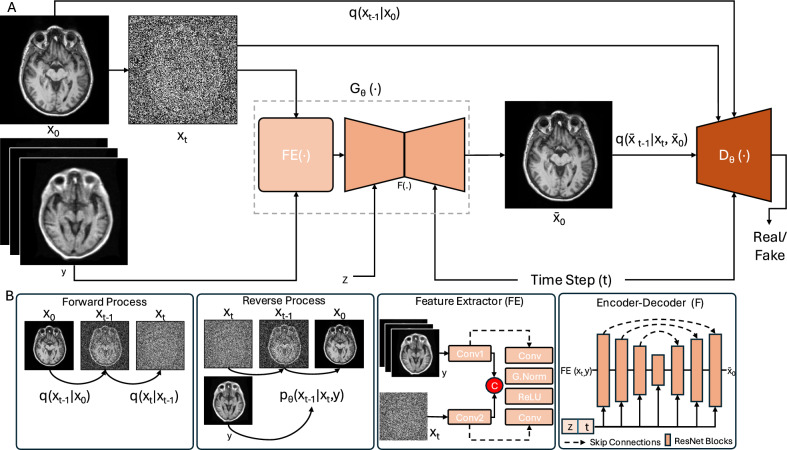
Overview of the proposed SynPoC Model. The figure illustrates the forward and reverse diffusion processes involved in translating PoC MRI images to high-field MRI images. In the forward process, Gaussian noise is progressively added to the high-field MRI image $x_0$, producing noisy image $x_t$ over time steps $t$. The reverse process involves a neural network $G_\theta (\cdot )$ that denoises the perturbed image using extracted features (via Feature Extractor, $FE(\cdot )$) and the encoder-decoder $F(\cdot )$ from both the conditional ULF MRI image ($y$) and noisy target contrast ($x_t$) to gradually reconstruct the high-field MRI image ($\tilde{x}_0$). The encoder-decoder network $F(\cdot )$ follows a U-Net-like architecture with skip connections, designed to capture high-resolution spatial features. The discriminator, $D_\theta (\cdot )$, is a time-dependent convolutional network that distinguishes between true $q(x_{t-1}|x_t,y,x_0)$ and estimated $q(\tilde{x}_{t-1}|x_t,y,\tilde{x}_0)$ distributions, focusing on high-frequency details and realism.

A fundamental assumption within the denoising diffusion process is that the distribution $p_\theta (x_{t-1}|x_t,y)$ can be effectively represented by a Gaussian distribution. This can be accomplished by employing sufficiently small noise variance ($\beta _t$) over thousands of larger steps ($T$), rendering the forward diffusion inherently Gaussian and consequently shaping the denoising distribution into a Gaussian form. However, as the number of steps increases, inference time substantially extends, and there is no assurance that the Gaussian assumption consistently holds, particularly with large step sizes. To address this challenge, the denoising distribution was modeled as a multimodal distribution with a reduced number of steps, using a GAN^59^ to approximate the true distribution. Inspired by this approach, SynPoC adopts a GAN-based adversarial diffusion approach to formulate the proposed network designed for high-field MRI synthesis as in Fig. 9.

#### Model components

SynPoC consists of a generator $G_\theta$ and a discriminator $D_\theta$ for modelling the denoising diffusion process. The $G_\theta$ consists of a Feature Extractor module $FE(\cdot )$ and the encoder-decoder structure $F(\cdot )$ as follows in Eq. (6).

6 $$\begin{aligned} \begin{aligned} G_\theta (\cdot )= FE(\cdot ) + F(\cdot ) \end{aligned} \end{aligned}$$

The feature module ($FE(\cdot )$) utilizes separate residual blocks ($Conv1$ and $Conv2$) for perturbed high-field MRI and conditional low-field MRI contrast. Each residual block comprises a convolutional layer followed by Group Normalisation (G.Norm), ReLU activation, and another convolutional layer. This approach enables the extraction of contrast-specific feature representations from both the conditional and target image contrasts. The concatenated features are then processed through $F(\cdot )$ to predict the denoised version of the noisy target contrast.

The generator $G_\theta$ is a 2.5D model that processes the noisy high-field MRI contrast $x_t$ and the low-field conditional contrast *y* with adjacent slices, along with a 256-dimensional latent vector *z* from a three-layer MLP. This vector modulates the feature maps through adaptive normalization during the reverse diffusion process, enabling $G_\theta$ to approximate the initial denoised high-field MRI contrast $x_0$, as outlined in Eq. (7). This 2.5D design balances computational efficiency and spatial consistency, avoiding the high memory demands of a fully 3D implementation while leveraging inter-slice information effectively.

7 $$\begin{aligned} \begin{aligned} \tilde{x}_0 = G_\theta (x_t,y,z,t) \end{aligned} \end{aligned}$$

The discriminator $D_\theta (\cdot )$ encourages the learned feature representation to align more closely with the distribution of the target contrast. It is a time-dependent discriminator that differentiates between $x_{t-1}$ and $x_t$ by deciding if $x_{t-1}$ is a plausible denoised version of $x_t$. To train the discriminator, we derive the denoised version of the predicted target contrasts at timestep $t-1$, which is $\tilde{x}_{t-1}$ using the posterior distribution (as in Eq. 8) and obtain the corresponding of the actual target contrast $x_{t-1}$ from the forward diffusion process (Eq. 1).

8 $$\begin{aligned} \begin{aligned} p_\theta (\tilde{x}_{t-1}|x_t,y)\sim q(x_{t-1}|x_t, \tilde{x}_0 = G_\theta (x_t,y,z,t)) \end{aligned} \end{aligned}$$

The discriminator is then trained by maximizing the objective function described in Eq. (9):

9 $$\begin{aligned} \begin{aligned} L_{D_{\theta }} & =\sum _{t\geqslant 1}^{}\mathbb {E}_{q (x_{t}|x_0,y)}[\mathbb {E}_{q(x_{t-1}|x_t,y)} [\eta \log (D_\theta (x_{t-1},x_t,t))] \\ & \quad + \mathbb {E}_{p_\theta (x_{t-1}|x_t,y)} [(1-\eta )\log (1-(D_\theta (\tilde{x}_{t-1},x_t,t)))]] \end{aligned} \end{aligned}$$

where $\eta =0$ if the $x_{t-1}$ is obtained from prediction of the SynPoC, and $\eta =1$ if the $x_{t-1}$ is derived from the actual target contrast distribution.

The generator $G_\theta$ is trained using minimizing the non-saturating adversarial loss^60^ and $L_1$ loss, as indicated in Eq. (10):

10 $$\begin{aligned} \begin{aligned} L_{G_{\theta }} = \sum _{t\geqslant 1}^{}\mathbb {E}_{p_\theta (x_{t-1}|x_t,y)}[-\log (D_\theta (\tilde{x}_{t-1},x_t,t))]+\lambda _1 L_1 (G_\theta (x_t,y,t,z), x) \end{aligned} \end{aligned}$$

where $\lambda _1=0.5$ acts as a hyperparameter that controls the contribution of the loss component.

### Model implementation

All models were developed using Pytorch and trained on two NVIDIA A40 GPUs, each with 46GB of memory. For training, the Adam optimizer was employed with initial learning rates of $1.5 \times 10^{-4}$ for the generator and $1 \times 10^{-4}$ for the discriminator, with momentum parameters $\beta _1 = 0.5$ and $\beta _2 = 0.9$, following the procedures recommended in^50^. The diffusion process utilized four steps with noise variance parameters set from $\beta _{min} = 0.1$ to $\beta _{max} = 20$.

### Image analysis approach

MRI images were segmented across different modalities using SynthSeg${^+}$^53^, a robust AI segmentation suite capable of handling diverse clinical imaging conditions without retraining^53^. This choice is substantiated by its proven effectiveness in large-scale clinical brain MRI dataset analyses, where traditional segmentation methods fail due to variability in scan quality and acquisition parameters. For clinical cases in MS and hemorrhage, the contrast adaptive segmentation tool for lesion segmentation was used^56^.

Numerous studies have identified specific brain regions that are clinically important due to their involvement in neurological disorders. Volumetric changes in these regions can provide insight into disease progression and severity, making them key targets for analysis in neuroimaging studies^61–68^. To quantitatively validate the volumetric measurements derived from different MRI modalities, Bland-Altman plots will be utilized. These plots assess the agreement and consistency of measurements between the high-field images, the original PoC images, and those enhanced by our SynPoC model.

### Image quality metrics

To objectively evaluate the performance of our diffusion-based enhancement model, unwanted background regions (T1 brain mask) were removed before calculating these metrics, and the results were averaged across the available MRI contrasts. We employed the following standard image quality metrics:

**Peak Signal-to-Noise Ratio (PSNR)**, which measures the ratio between the maximum possible power of a signal and the power of corrupting noise. It is expressed in decibels (dB) and calculated as Eq. (11):

11 $$\begin{aligned} PSNR = 20 \cdot \log _{10}\left( \frac{MAX_I}{\sqrt{MSE}}\right) , \end{aligned}$$

where $MAX_I$ is the maximum possible pixel value of the image, and $MSE$ is the mean squared error between the enhanced and the original high-field images.

**Structural Similarity Index Measure** (SSIM), which is used to measure the similarity between two images. SSIM is defined as Eq. (12):

12 $$\begin{aligned} SSIM(x, y) = \frac{(2\mu _x \mu _y + c_1)(2\sigma _{xy} + c_2)}{(\mu _x^2 + \mu _y^2 + c_1)(\sigma _x^2 + \sigma _y^2 + c_2)}, \end{aligned}$$

where $x$ and $y$ are the windowed patches of the original and the enhanced image, $\mu$ and $\sigma$ are their average and variance, $\sigma _{xy}$ is the covariance, and $c_1, c_2$ are constants to stabilize the division.

**Mean Absolute Error (MAE)**, which quantifies the absolute difference between the enhanced and the original images (Eq. 13):

13 $$\begin{aligned} MAE = \frac{1}{N} \sum _{i=1}^{N} |x_i - y_i|, \end{aligned}$$

where $x_i$ and $y_i$ are the pixel values of the original and enhanced images, respectively, and $N$ is the total number of pixels.

## Supplementary Information

Supplementary Information.

## Data Availability

All data needed to evaluate the conclusions in the paper are present in the paper and/or the Supplementary Materials. To facilitate easier slice-by-slice comparison of critical results, we have also included video demonstrations that show side-by-side axial views across PoC, SynPoC, and 3T images. These videos are available as part of the Supplementary Materials and can be accessed through our GitHub repository: https://github.com/BioMedAnalysis/SynPoC. The repository contains both the source code of SynPoC and synchronized axial comparison videos that correspond to the key experiments and to Supplementary Figures S2–S4 to aid slice-by-slice inspection. Access to the original MRI datasets and associated annotations is restricted due to patient privacy obligations and institutional regulations. These datasets, which include both internal and external sources, were obtained with the necessary institutional permissions and IRB (Institutional Review Board) approval, and are therefore not publicly available. However, interested researchers may request access to the data supporting the findings of this study for non-commercial and academic purposes by contacting the corresponding author directly.
