# Supplementary material for: SynPoC: a high-quality generative diffusion model for transforming ultra-low-field point-of-care MRI using high-field MRI representations
Source: Sci Rep. 2026 Jan 24;16:3285. doi: 10.1038/s41598-025-33162-9 (PMC12835117; doi:10.1038/s41598-025-33162-9)
Supplement: Supplementary file 1 — Supplementary Information. [file 41598_2025_33162_MOESM1_ESM.pdf]

SynPoC: A High-Quality Generative Diffusion Model for Transforming Ultra-Low-Field Point-of-Care MRI Using High-Field MRI Representations

Kh Tohidul Islam[1,2,+], Sanuwani Dayarathna[11,+], Shenjun Zhong[1], Parisa Zakavi[1], Helen Kavnoudias[3,13], Shawna Farquharson[2], Gail Durbridge[5], Hongfu Sun[6], Stephen Bacchi[14], Gary F. Egan[1], Markus Barth[6], Andrew Dwyer[7,12], Katie L. McMahon[8], Paul M. Parizel[9,10], Meng Law[3,4], and Zhaolin Chen[1,11,*]

[1]Monash Biomedical Imaging, Monash University, Blackburn Road, Clayton, Victoria 3168, Australia
[2]Australian National Imaging Facility, Queensland, Australia
[3]Department of Neuroscience, Monash University, Clayton, Victoria, Australia
[4]Radiology, Alfred Hospital, Victoria, Australia
[5]Herston Imaging Research Facility, University of Queensland, Queensland, Australia
[6]School of Electrical Engineering and Computer Science, University of Queensland, Queensland, Australia
[7]South Australian Health and Medical Research Institute, South Australia, Australia
[8]School of Clinical Science, Queensland University of Technology, Queensland, Australia
[9]David Hartley Chair of Radiology, Royal Perth Hospital, Western Australia, Australia
[10]Medical School, University of Western Australia, Western Australia, Australia
[11]Data Science and AI, Monash University, Exhibition Walk, Clayton, Victoria 3800, Australia
[12]SA Medical Imaging, SA Health, South Australia, Australia
[13]Surgery, Monash University, Clayton, Victoria, Australia
[14]Department of Neurology, Royal Adelaide Hospital, South Australia, Australia
[*]zhaolin.chen@monash.edu
[+]these authors contributed equally to this work

ABSTRACT

Supplementary Material

Supplementary A: Comparison of SynPoC and other models

To evaluate the performance of the SynPoC model, we compared its results against three state-of-the-art generative models: Pix2Pix, pGAN, DDPM, and 3D U-Net, in both quantitative and qualitative assessments. The comparison focuses on the synthesis of high-field T2 MRI contrasts using the SITE-1 healthy dataset, employing the same train-test split as described in Section 4. As shown in Table S1, SynPoC outperforms all other models, surpassing the GAN-based models by up to 1.61dB in PSNR, 29% in SSIM, and 10% in MAE. The superior performance of SynPoC can be attributed to its ability to effectively model complex tissue structures and intensity variations, leading to a more accurate and artifact-free synthesis of T2 MRI contrasts. We also compared SynPoC with a 3D U-Net baseline to demonstrate the effectiveness of our model against 3D architectures. For a fair comparison, we extracted 2D axial slices from the synthesized outputs of the 3D models for quantitative evaluation. The results clearly show that even with 3D modelling, the performance falls short of SynPoC's results.

Table S1. Quantitative performance metrics (PSNR, SSIM, MAE) for T2 MRI contrasts from SITE-1, comparing SynPoC with other baselines (without any mask).

	Pix2Pix	pGAN	DDPM	3D U-Net	SynPoC
**PSNR**	21.17±1.28	20.64±0.98	20.42±0.87	22.06±1.08	**22.25±1.42**
**SSIM**	0.76±0.06	0.76±0.05	0.52±0.06	0.76± 0.06	**0.81±0.05**
**MAE**	0.25±0.03	0.26±0.03	0.31±0.04	0.32±0.04	**0.21±0.03**

Figure S1 provides a visual comparison that highlights SynPoC's superior synthesis quality, exhibiting higher fidelity and fewer artifacts in the generated images. In contrast, the GAN-based methods, such as Pix2Pix and pGAN, display more artifacts and lower tissue depiction accuracy, while DDPM results in blurriness and significant information loss, leading to lower SSIM scores. The synthesis results from the 3D U-Net also exhibit increased blurriness and artifacts, result in lower accuracy.

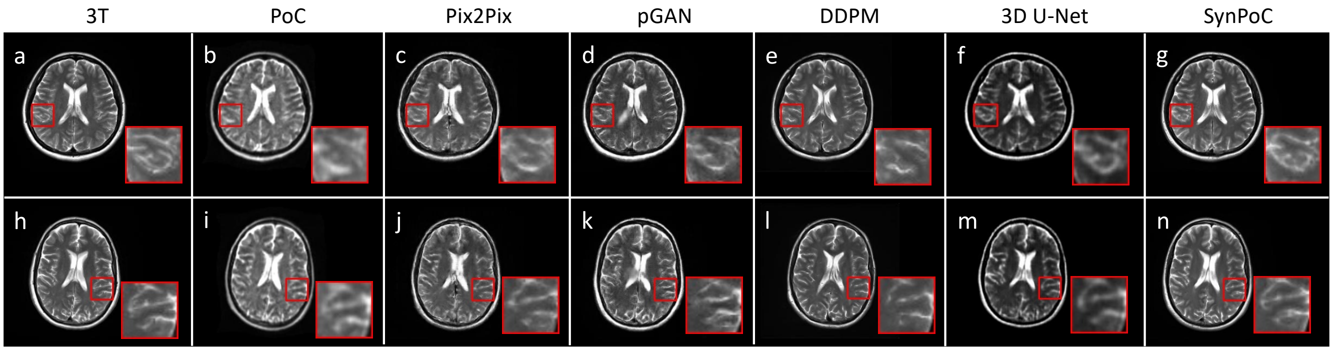

Figure S1. Qualitative comparison of synthesized T2 MRI contrasts for healthy participants from the SITE-1 dataset. Each row presents samples generated by different models and their corresponding error maps. (a) and (h) PoC images, (b) and (i) 3T images, (c) and (j) Pix2Pix images, (d) and (k) pGAN images, (e) and (l) DDPM images, (f) and (m) 3D U-Net images, (g) and (n) SynPoC images.

Supplementary B: Visualization and comparison of 3T, PoC, and SynPoC 3D image volumes.

In this section, we provide comprehensive visual comparisons between the high-field 3T MRI images, the original ultra-low-field PoC MRI images, and the SynPoC-enhanced images. The figures below display montages of 100 axial slices from each modality, arranged in a 10x10 grid. These montages are intended to demonstrate the overall image quality, anatomical detail, and the enhancements achieved by the SynPoC model across a large dataset.

Figure S2 presents a montage of axial slices from the 3T MRI scans. These images serve as the reference standard, showcasing the high image quality and detailed anatomical features that are achievable with high-field MRI systems. The images display a clear delineation of brain structures, including cortical folds, subcortical nuclei, and ventricular systems.

Figure S3 displays a montage of axial slices from the PoC MRI scans. These images highlight the challenges associated with ultra-low-field MRI, such as lower spatial resolution, reduced contrast, and increased noise. The anatomical structures are less distinct, making it more difficult to identify fine details that are important for clinical assessments.

Figure S4 shows a montage of the SynPoC-enhanced images corresponding to the PoC MRI slices presented in Figure S3. The SynPoC model significantly improves the image quality, enhancing contrast and reducing noise, resulting in images that closely resemble the high-field 3T MRI scans (Figure S2). The anatomical structures become more discernible, with better definition of cortical and subcortical regions, thereby enhancing the potential for accurate clinical interpretation.

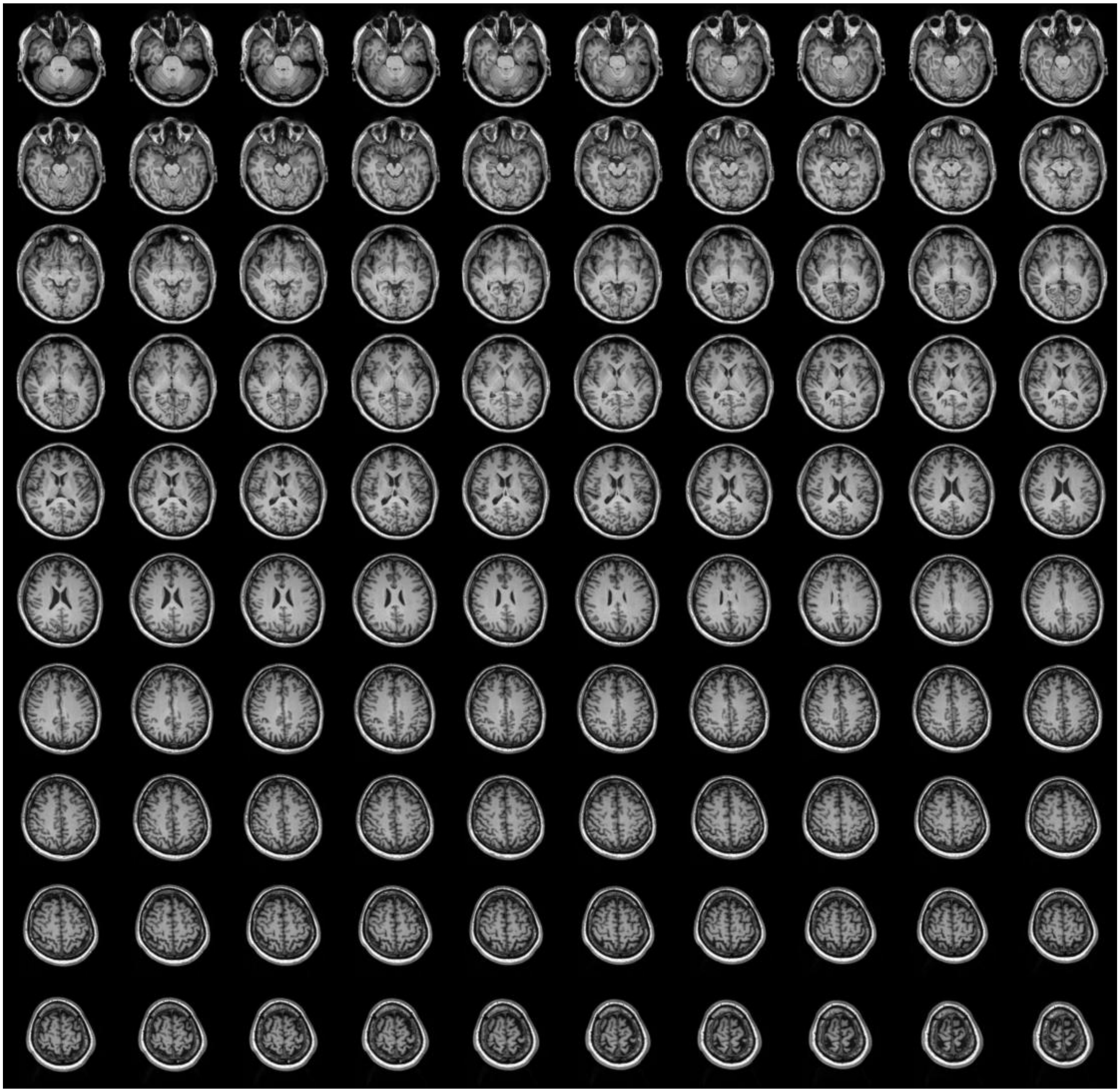

Figure S2. Montage of 100 axial slices from high-field 3T MRI scans. The images are arranged in a 10x10 grid, displaying the high-resolution and detailed anatomical structures typical of 3T MRI imaging.

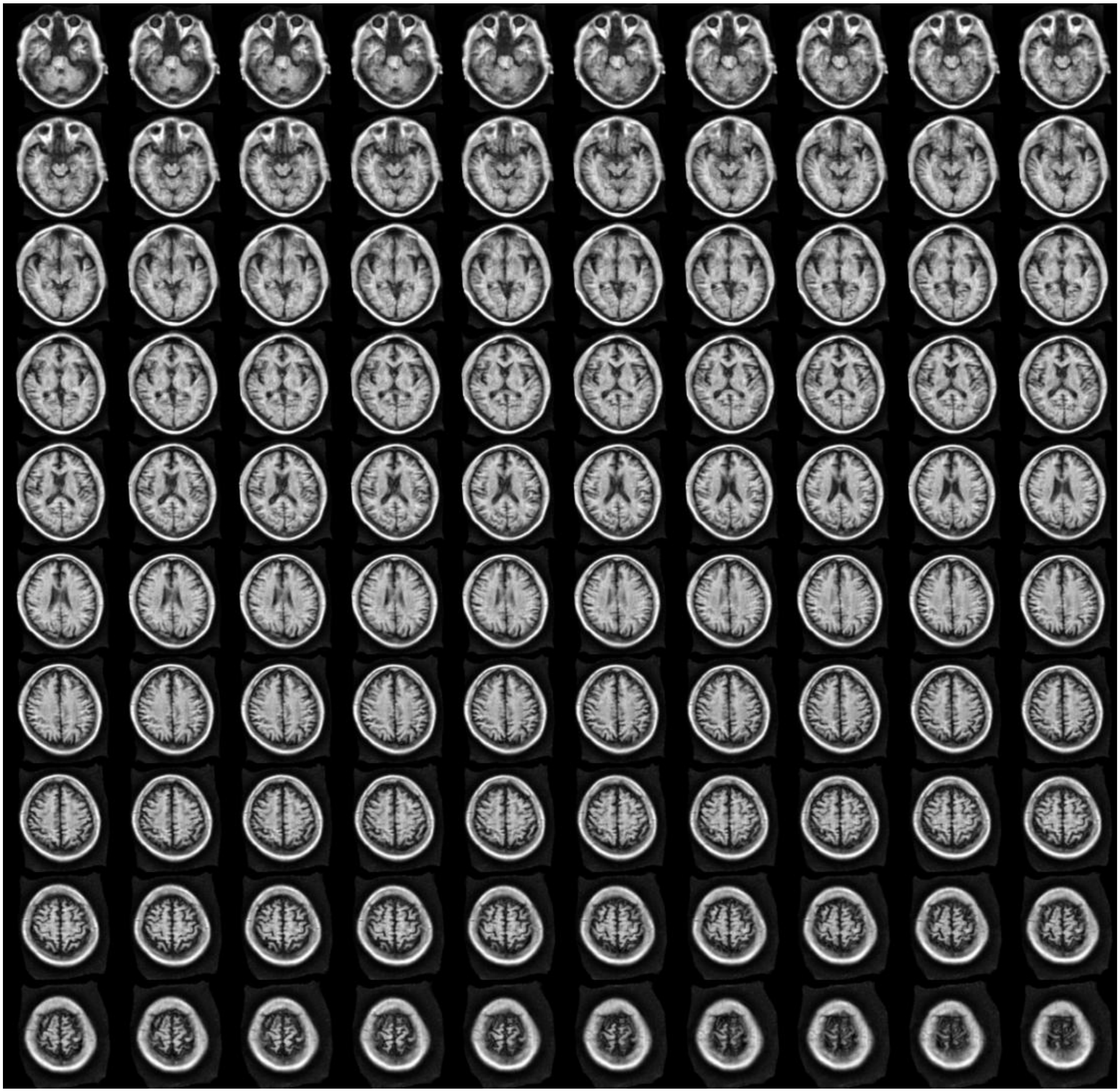

Figure S3. Montage of 100 axial slices from ultra-low-field PoC MRI scans. The images are arranged in a 10x10 grid, illustrating the reduced image quality and anatomical detail due to lower field strength and signal-to-noise ratio.

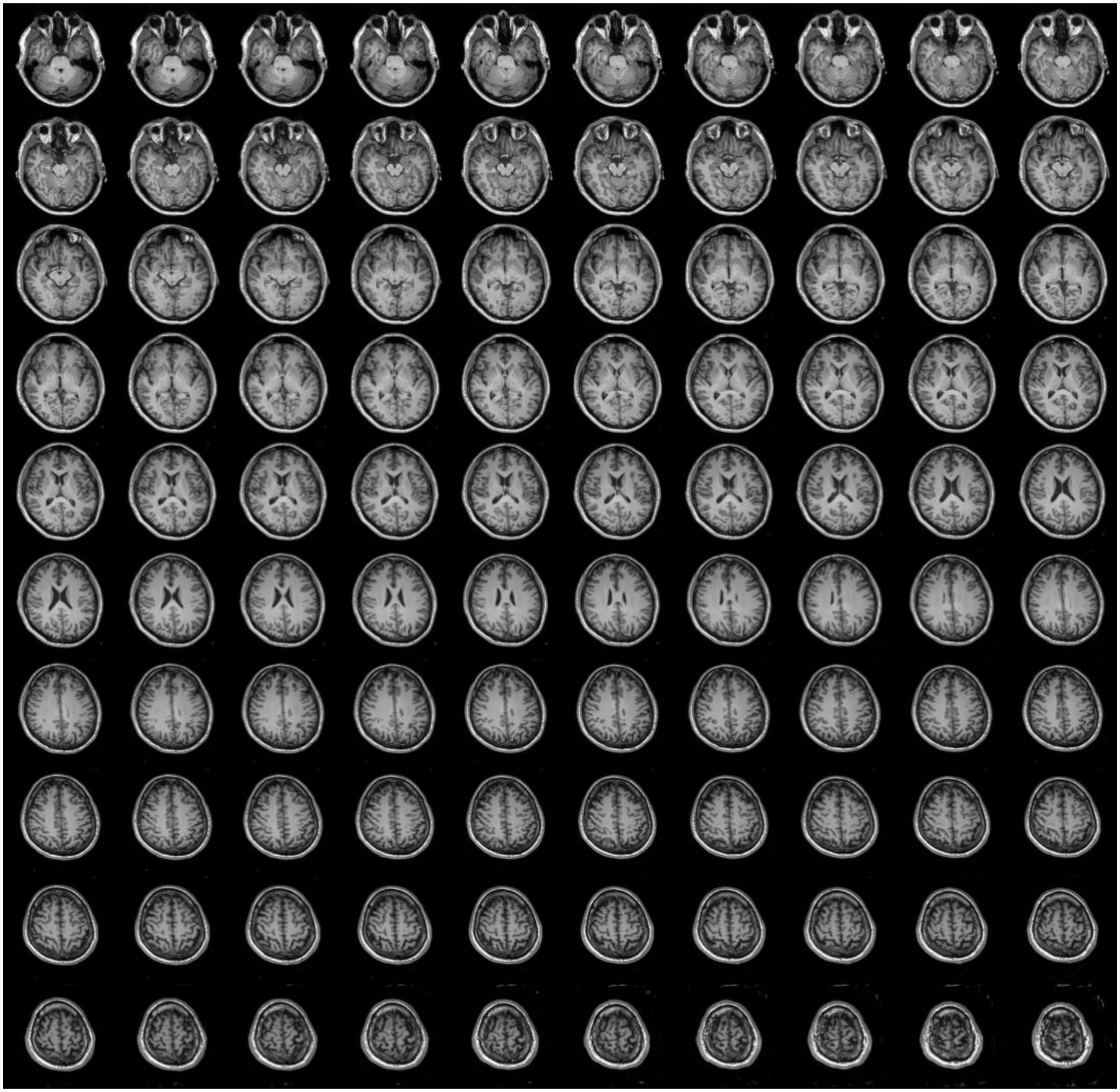

Figure S4. Montage of 100 axial slices from SynPoC-enhanced MRI images. The images are arranged in a 10x10 grid, demonstrating the improvements in image quality and anatomical detail achieved by the SynPoC model, closely approximating high-field MRI images.
